# Association of cardiac NT pro-β-type natriuretic peptide with metabolic and endothelial risk factors in young obese hypertensive patients: a perspective on the hypothalamic pituitary adrenal axis activation

**DOI:** 10.1186/s13098-016-0164-2

**Published:** 2016-07-29

**Authors:** Mona Schaalan, Waleed Mohamed, Rania Rahmo

**Affiliations:** 1 Department of Biochemistry and Clinical Pharmacy and Pharmacy Practice, Faculty of Pharmacy, Misr International University, Km 28, Cairo-Ismailia Road, Cairo, Heliopolis, PO Box 1, Cairo, Egypt; 2 Chemistry Department, Cairo General Hospital, Cairo, Egypt; 3 Pharmacology and Toxicology Department, Misr International University, Cairo, Egypt

**Keywords:** Cortisol, Aldosterone, NT-proBNP, Endothelial markers, Hypertension, Metabolic syndrome

## Abstract

**Background:**

In practice, there is increasing recognition of the importance of hypothalamic pituitary adrenal axis in the cardiovascular disease progression. The association of brain natriuretic peptide with obesity and characteristics of the metabolic syndrome in adults and aged patients is well established, but that in pediatrics needs thorough elucidation.

**Aim:**

The aim of this study was to assess the association of hypothalamic pituitary adrenal axis mediators (cortisol and aldosterone) with plasma NT-pro β-type natriuretic peptide (NT-proBNP) levels on metabolic, immune-inflammatory and endothelial markers in young obese pediatric patients.

**Methods:**

This is achieved by recruitment of 60 young (13–17 years) obese pediatric cohorts who are further subclassified according to their stage of hypertension; normotensive, prehypertensive and hypertensive patients.

**Results:**

The study showed significant differences in the metabolic parameters (glucose, insulin and HOMA-index) among the three obese young patient groups. Levels of cortisol and aldosterone, as well as NT-proBNP levels are positively associated with characteristics of the metabolic syndrome; blood pressure, BMI, HOMA index in all three obese groups. However, their association to the lipid profile was insignificant. These increases aligned harmonically with the assessed immune-inflammatory markers; CRP, TNF-α, and IL-23, as well as levels of sICAM, sVCAM and p-selectin, reflecting the involvement of mast cells and inflammatory effects on the vascular endothelium. ROC analysis revealed their beneficial addition as promising biomarkers for a better prognostic profile of hypertension-induced cardiovascular risk.

**Conclusion:**

Early detection of NT-proBNP, cortisol and aldosterone levels in pre-hypertension stage added to the immune-inflammatory mediators may improve the coronary risk assessment in young Egyptian patients.

## Background

Despite the high prevalence of hypertension in the adult population, identification of its key determinants remains arduous due to its multifaceted and polygenic nature. Because of to the striking epidemic state of childhood obesity, a growing number of obese adolescents are now at risk for this silent disease. Therefore, the medical recommendations are directed towards early detection of pre-hypertension (pre-HT) stages and actively targeting lifestyle patterns with various risk reduction measures in such young population. Early diagnosis of pre-HT stage serves as an early alarming manifest of certain metabolic derangements that will ultimately lead to metabolic syndrome (MS) and cardiovascular diseases. The association of obesity with the characteristics of the metabolic syndrome, and many other metabolic risk factors in adults and aged patients, is well established but that in pediatrics needs thorough elucidation.

As obesity and the metabolic syndrome are known to be related to a state of chronic low grade inflammatory stress, it is hypothesized that this causes a stimulatory response to hypothalamic adrenal axis (HPA) together with the sympathetic nervous system; events that create a state of clinically silent hypercortisolism. The latter phenomenon is associated with excessive cortisol, aldosterone and ACTH production [1].

Mineralocorticoid receptor (MR) has high binding affinity for both cortisol and aldosterone [1], whereas glucocorticoid receptor (GR) is selective for cortisol. Aldosterone promotes MR, which in turn may be followed by inflammation and vascular injury, ultimately leading to heart and renal diseases as well as stroke. Augmented levels of these hormones in hypertensive individuals may be associated with ACTH secretion which enhances the production of not only cortisol, but also of aldosterone for a short-term period. Individuals with less efficient cortisol synthesis maintain a subtle ACTH drive to the adrenal gland which may result in hyperplasia of both zona fasciculata and glomerulosa, causing increased synthetic capacity for both cortisol and aldosterone [2].

Chronically activated HPA axis is also linked to higher prevalence of insulin resistance, obesity, IGT, hypertension and dyslipidemia. This metabolic derangement is due to activated adipocytokine release which in turn results in a state of low level systemic chronic inflammation inducing atherosclerosis and endothelial dysfunction [3, 4].

Beta-type natriuretic peptide (BNP), a neurohormone synthesized in the cardiac ventricles, is released as pre proBNP and then enzymatically cleaved to the *N*-terminal-proBNP (NT-proBNP) and BNP upon ventricular myocyte stretch. In practice, there is increasing awareness to the significance of BNP in the pathophysiology, diagnosis and prognosis of cardiovascular disorders beyond heart failure [5]. The association of BNP to obesity and characteristics of the metabolic syndrome in adults and aged patients is well established, but that in pediatrics needs scrupulous elucidation. Albeit the well-documented associations between natriuretic peptide levels and obesity, information on potential links with other metabolic risk factors and adrenal hormones are still contentious.

Vascular endothelial dysfunction is known to occur early in the atherosclerotic process and is predictive of cardiovascular prognosis. Cardiovascular risk factors such as hypertension, diabetes, hyperlipidemia, and obesity have been observed to be associated with vascular endothelial dysfunction, even prior to the development of clinically evident coronary atherosclerosis [6]. Indeed, vascular endothelial dysfunction can be stimulated by inflammatory triggers that include free radicals and proinflammatory cytokines, producing adhesion molecules such as intercellular adhesion molecule-1 (ICAM-1), p-selectin and vascular cell adhesion molecule-1 (VCAM-1), vasoactive peptides such as endothelin-1; generation of CRP, all of which are involved in the modulation of leukocyte recruitment (mediating the attachment and transmigration of leukocytes across the endothelial surface) and platelet adhesion [7]. Interestingly, excess glucocorticoids are reported to impair endothelium-dependent vasodilatation in humans in vivo [8].

The aim of this study was to assess the association of plasma NT-pro β-type natriuretic peptide (NT-proBNP) levels and mediators of HPA axis; cortisol and aldosterone in young obese metabolic syndrome patients with escalating stages of hypertension; normotensive (NT), pre-hypertension (pre-HT) and hypertensive (HT) pediatric patients. Moreover, the effects on metabolic risk factors, pro-inflammatory cytokines; TNF-α, IL-6 and IL-23, as well as vascular endothelial markers; ICAM, VCAM and p-selectin are addressed.

## Methods

In the current non-randomized study, 60 obese patients were enrolled. These comprised 20 obese normotensive, 20 prehypertensive and 20 stage 1 hypertensive young patients from the Pediatric Outpatient Clinic at the Endemic Disease Hospital at Cairo University in Egypt. The average age was 13–17 years, including both genders (27 males and 33 females). *Group I (obese-NT)* was composed of 20 young obese normotensive patients with MS (11 females and 9 males, mean age 13.8 ± 0.9 years). *Group II (MS-pre-HT)* included 20 young obese hypertensive patients (11 females and 9 males, mean age 14.7 ± 1.6 years); while *Group III (MS-stage 1HT)* included 20 young obese patients diagnosed with stage 1 hypertension (11 females and 9 males, mean age 15.1 ± 1.1). Thirty volunteers (age and sex matched; 12 females, 18 males) with normal BP (<120/80 mmHg), healthy hemodynamic and normal biochemical parameters were recruited in our study as a healthy control group.

### Ethics approval and consent to participate

The study protocol was approved by the local ethics committee of the Faculty of Medicine, Cairo University, and informed written consent was obtained from the parents of the patients and volunteers before entering the study.

*Inclusion criteria* for both groups were pediatric age (≤18 years) when diagnosed with obesity, described as BMI > 30. The classification of the stage of hypertension was determined according to JNC-8 as follows;

*Normal control* systolic BP < *120* mmHg and diastolic BP: <*80* mmHg.

*Pre-HT* systolic BP: *120–139* mmHg and/or diastolic BP: *80–89* mmHg.

*Stage1 HT* systolic BP: *140–159* mmHg and/or diastolic BP: *90–99* mmHg.

*According to the criteria of the US National Cholesterol Education Program Adult Treatment panel III (2001), metabolic syndrome was diagnosed if at least three of the following elements were fulfilled including central obesity;*

- Central obesity [waist circumference ≥102 (men), ≥88 cm (women)].
- Dyslipidemia; elevated serum triglyceride levels ≥1.7 mmol l/L (150 mg/dL), reduced serum HDL-cholesterol <40 mg/dL(men), <50 mg/dL) (women).
- Elevated fasting plasma glucose ≥6.1 mmol l/L(110 mg/dL).
- Elevated blood pressure (BP, systolic BP ≥ 130 mmHg or diastolic BP ≥ 85 mmHg).

*Exclusion criteria* referred to autoimmune disease states, acute kidney injury or unsatisfactory vascular access or any other known condition that would affect cytokine levels. None of our patients had been treated with antibiotics, anti-inflammatory or corticosteroid medications during the study period.

Venous blood samples (2 ml) were obtained early in the morning after overnight fasting from all patients/controls and were divided into two aliquots: one part was anticoagulated for plasma separation for NT-proBNP assessment. The remainder of the samples were allowed to clot and sera were then separated by centrifugation (3500 rpm, 20 min, 25 °C) and stored at −20 °C for later biochemical determinations. Serum levels of fasting glucose, insulin and lipid profile (T-Chol, HDL-C and TG) were analyzed using Synchron CX5 autoanalyzer (Beckman, USA), while LDL-cholesterol levels were calculated by using the Friedewald formula.

Moreover, pro-inflammatory markers such as tumor necrosis factor (TNF)-α, interleukin (IL)-6 and interleukin IL-23 were assessed using commercially available enzyme-linked immunosorbent assay kits (R&D Systems Inc., Minneapolis, MN, USA) according to the manufacturer instructions. The intra-/inter- assay coefficient of variation (CVs) for TNF-α is 10–12 %, and its lower level of detection (LOD) was 3 pg/ml, while the LOD for IL-6 = 1 pg/ml and IL-23 = 0.2 pg/ml. High sensitivity CRP was assessed using available enzyme-linked immunosorbent assay kits (R&D Systems Inc., Minneapolis, MN, USA) according to the manufacturer instructions. The intra-/inter- assay coefficient of variation (CVs) for TNF-α is 10–12 %, and its LOD = 0.1 mg/L.

Serum levels of soluble adhesion molecules; sICAM, sVCAM, p-selectin were determined using commercial monoclonal antibody-based enzyme-linked immunosorbent assay (ELISA) kits (Ray-Bio^®^ Human, RayBiotech, Inc., Norcross, GA). The intra-/inter- assay coefficient of variation (CVs) for serum p-selectin is 10–12 %, whereas LOD were as follows: p- selectin = 30 pg/ml, sVCAM = 0.3 ng/ml and sICAM = 23 pg/ml.

In the separated plasma portions, the level of NT-proBNP was quantitatively assessed using commercial monoclonal antibody-based enzyme-linked immunosorbent assay (ELISA) kits (COBAS from Roche diagnostics, GmbH, Mannheim, Germany), with LOD = 5.00 pg/ml. Aldosterone, ACTH and cortisol were measured by RIA technique (Invitrogen, California, USA).

Measurements were performed according to the manufacturer’s instructions; each sample was measured in duplicate and the arithmetic mean was considered as a final result. Results were calculated by reference to standard curves.

*Clinical assessments* included complete history taking, past medical and disease history for confirming the appropriateness of the patients to the inclusion criteria. BP and BMI monitoring were according to the international guidelines.

### Statistical analysis

Continuous variables are expressed as mean ± standard deviation (SD) and normality was assessed by the Shapiro–Wilk test. Normal transformation of non-normally distributed data was done by Lg10 multiplication. Differences between groups were assessed by one-way analysis of variance (ANOVA) followed by Tukey–Kramer post hoc test. The association between the parameters was determined using the Pearson’s correlation coefficient. Receiver operating characteristic (ROC) curve analysis was used to assess the predictive ability of the assessed biomarkers; the area under the curve (AUC) and the confidence intervals (CI) were calculated with the Wilcoxon and Mann–Whitney tests. All reported probability values were two-tailed, and a P value <0.05 was considered statistically significant. Statistical analysis was performed using the SPSS 20.0 statistical software package (SPSS Inc., Chicago, Ill., USA).

## Results

Characteristics of the study participants are listed in Table 1; their age range was 13–17 years. Overall, 20 young obese normotensive young patients (11 females and 9 males), as well as 60 young obese patients with BMI exceeding 30 (33 females and 27 males) were enrolled in the study. According to the JNC-8 classification these obese patients were further subclassified according to their stage of blood pressure; normotensive (NT), prehypertensive pre-HT and stage 1 hypertension (HT).

**Table 1:** Anthropometirc measures of obese patients with NT, pre-HT and HT and their plasma metabolic parameters (vs their control group)

	Control-patients (N = 30)	Obese patients		
		NT (N = 20)	Pre-HT (N = 20)	HT (N = 20)
Age (years)	14.5 ± 1.5	13.8 ± 0.9	14.7 ± 1.6	15.1 ± 1.1
Gender (M/F)	18/12	9/11	9/11	9/11
Diastolic BP (mmHg)	72. 5 ± 5.2	80.3 ± 2.1*	83.1 ± 2.5^*^	87.3 ± 2.6*^, # a^
Systolic BP (mmHg)	114 ± 6.1	117.5 ± 3.4	128.1 ± 3.9*^,^^#^	147 ± 6.5*^, #, a^
BMI	21.2 ± 1.2	32.9 ± 0.5*	32.2 ± 0.7^*^	33.6 ± 0.6*
Glucose (mg/dl)	78.2 ± 4.7	105.4 ± 3.3*	113.6 ± 1.8*^, #^	125.6 ± 5.3*^,#, a^
Insulin (µIU/ml)	5.5 ± 0.7	8.5 ± 0.4*	10.5 ± 1.5*^, #^	13.5 ± 0.7*^, #, a^
HOMA-index	1.1 ± 0.1	2.2 ± 0.03*	2.9 ± 0.06*	4.1 ± 0.09*^, #, a^
TG (mg/dl)	75.8 ± 10.4	198.7 ± 19.7*	206.5 ± 28.9*	215.9 ± 22.5*
TC (mg/dl)	132.1 ± 7.2	289.2 ± 16.7*	299.2 ± 30.7*	312.5 ± 21*^, #^
LDL-C (mg/dl)	77.2 ± 4.7	199.6 ± 12.8*	208. 5 ± 23.9*	218.9 ± 13.7*^, #^
HDL-C (mg/dl)	39.73 ± 3.5	49.75 ± 3.8*	49.46 ± 4.1*	50.53 ± 3.8*
Metabolic syndrome	No	No	Yes	Yes

Values are mean ± SD for control healthy, obese normotensive, prehypertensive and hypertensive patients groups

Values (*) is significantly different from control group; (^#^) significantly different from normotensive obese patients, (^a^) significantly different from prehypertensive obese patients

Values are statistically significant at *P* < 0.05 using one way ANOVA with LSD as post hoc test. (SPSS program)

The increase in the assessed metabolic parameters; glucose, insulin and HOMA-index has progressed in accordance with the stage of blood pressure; data shown in Table 1. Both glucose and insulin levels in normotensive obese group increased significantly approximately 1.34, 1.53 fold; respectively, compared to control group. The significant elevation of glucose level in the obese prehypertensive and hypertensive groups reached 1.45 and 1.6 folds, respectively from the baseline control value. Moreover, the significant increase of obese pre-HT reached 7.7 % from the obese NT, while the extent of elevation of obese HT reached 10.5 % times from pre-HT and 19.1 % from NT; all significant at P < 0.05.

A similar pattern was obvious in the insulin level, where the significant elevation of insulin level in the obese prehypertensive and hypertensive groups reached 1.9 and 2.42 folds, respectively from their baseline control value. Moreover, the significant increase of obese pre-HT reached 23.9 %; from the obese NT, while the extent of elevation of obese HT reached 27.5 % times from pre-HT and 58 % from NT; all significant at P < 0.05. The HOMA-index doubled in the obese NT (2.21 vs 1.06), increased 33.4 % in the pre-HT group (2.95), and 41 % in the obese HT group (4.16).

Concerning the lipid profile, illustrated in Table 1, the significant elevation was only reported in the obese NT group, as the levels of TG, T-Chol and LDL-C increased 2.6, 2.2 and 2.58 folds, respectively, compared to their normal control values (75.8 ± 10.37; 132.1 ± 7.16, 77.2 ± 4.7; respectively). The tracked increase of the aforementioned levels in the pre-HT and HT groups was subtle, yet insignificant; except for T-Chol and LDL-C in the HT group, where their increase was significant and reached 8 and 9.6 %, respectively, compared to their obese NT levels. The change of HDL-C concentration among the three obese groups (NT, pre-HT and HT) was undetectable, however they were all significantly higher than the normal levels (1.25,1.24, 1.27 fold, respectively).

Table 2 illustrates the progressive elevation of NT-proBNP and the adrenal hormones levels, cortisol, aldosterone and ACTH from obese normotensive to hypertensive individuals.

**Table 2:** Serum adrenal hormones, endothelial, and inflammatory markers in control, obese patients with NT, pre-HT & HT

	Control-subjects (N = 30)	Obese patients		
		NT (N = 20)	Pre-HT (N = 20)	HT (N = 20)
*Adrenal hormones*				
Cortisol (µg/dL)	9.1 ± 1.4	5.6 ± 0.7*	8.9 ± 2.4*^, #^	12.7 ± 0.5*^, #, a^
Aldosterone(µg/dL)	88.6 ± 8.5	132.1 ± 6.9*	195.9 ± 70.9*^, #^	320.2 ± 22.4*^,#, a^
ACTH(pg/ml)	23.8 ± 2.9	24.8 ± 5.6	79.6 ± 42.07*^, #^	44.2 ± 7.5*^, #, a^
*NT-pro BNP(pg/ml)*	73.07 ± 11.05	377.3 ± 87.8*	585.7 ± 155.7*^, #^	881.1 ± 130.9*^, # a^
*Cytokine markers*				
TNF-α (ng/ml)	2.3 ± 0.5	6.02 ± 1.4*	14.3 ± 7.6*^, #^	31.6 ± 6.7*^, #, a^
IL-6 (ng/ml)	1.2 ± 0.2	5.1 ± 1.2*	9.3 ± 4.6*^, #^	18.5 ± 2.4*^, #, a^
IL-23 (ng/ml)	16.1 ± 3.1	76.9 ± 6.3*	93.5 ± 15.5*^, #^	186.6 ± 23.5*^, #, a^
*hs-CRP (mg/L)*	0.3 ± 0.09	0.9 ± 0.12*	1.8 ± 0.9*^, #^	3.7 ± 0.8*^, #, a^

Values are mean ± SD for control healthy, obese normotensive, prehypertensive and hypertensive patients groups

Values (*) is significantly different from control group; (^#^) significantly different from normotensive obese patients, (^a^) significantly different from prehypertensive obese patients. Values are statistically significant at P < 0.05 using one way ANOVA with LSD as post hoc test. (SPSS program)

Regarding cortisol level, a significant decrease (37.9 %) in the obese NT group was observed, which is progressively elevated in both pre-HT and HT groups (1.6 and 1.4 times, respectively). The highest level of cortisol was recorded in the obese HT group (12.71 ± 0.553), which increased 42.7 % from the pre-HT level and is 2.3 times the obese NT level; all significant at P < 0.05.

Aldosterone level was significantly increased in the obese NT group (1.5 times) compared to the control level, and is further elevated in the pre-HT group (48.3 %). The HT level of aldosterone was the highest (320.2 ± 22.48) recording 1.63 folds the pre-HT level and 2.42 times the obese NT level, all significant at P < 0.05.

The effect of obesity on the ACTH normal level was undetectable, however the highest level was traced in the pre-HT group, which was 3.2 fold the obese NT value. Interestingly, ACTH levels decreased significantly (55.3 %) in the obese HT group.

The NT-proBNP levels in normotensive obese group increased significantly, approximately 5.2 fold (P < 0.05), compared to control group. The significant elevation in the obese prehypertensive and hypertensive groups reached 8 and 12 folds, respectively from baseline control value. Moreover, the significant increase of obese pre-HT reached 1.5 times from the obese NT; while the extent of elevation of obese HT reached 1.5 times from pre-HT and 2.3 times from NT; all significant at P < 0.05.

The levels of the assessed proinflammatory cytokine markers; TNF-α, IL-6 and IL-23 as well as CRP in all groups are presented in Table 2. It shows significantly increased levels of TNF-α, IL-6 and IL-23 and CRP by 2.6, 4.2, 5.9 and 2.3 times, respectively (P < 0.01), in obese normotensive patients, when compared with control healthy individuals group. The progressive elevation of inflammatory markers aligned with the stage of HT, as follows; the level of TNF-α in the pre-HT group was 2.4 times the obese NT level, and increased 2.2 times in the HT group; the latter is 5.25 fold the NT level, all significant at P < 0.05.

In the pre- HT group IL-6, IL-23 and CRP increased significantly 1.8, 1.2 and 2 fold, respectively, compared to the obese NT level. The levels of CRP and both cytokines; IL-6 and IL-23, doubled in the HT group, which reached 3.6, 2.42 and 4.2 fold the NT level, all significant at P < 0.05.

**Fig. 1:**
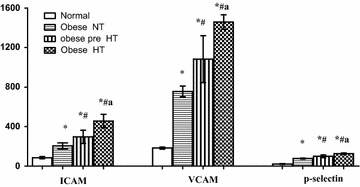
Pattern of adhesion molecules in the investigated groups (obese NT, obese pre-HT, obese HT) vs control. Values (*) is significantly different from control group; (^#^) significantly different from normotensive obese patients, (^a^) significantly different from prehypertensive obese patients

The endothelial markers, ICAM, VCAM and p-selectin, are illustrated in Fig. 1, showing 2.39, 4.13 and 3.58 fold significant increases in their levels in the obese NT, compared to their control levels. The increase in the levels of the endothelial markers among the obese groups was in alignment with the stage of hypertension. In the pre- HT group the increase in levels of ICAM, VCAM and p-selectin was 44.8, 43.2 and 30.5 %, respectively, compared with obese NT. These values were further elevated in the obese HT, as 53.6, 34.6 and 26.1 %, compared to obese pre-HT group, and 122.6, 92.8 and 64.5 %, compared to obese NT group; all respective to ICAM, VCAM and p-selectin levels.

### Correlational analyses between cortisol, aldosterone, NT-proBNP and hs-CRP with the metabolic and endothelial risk factors

Results of the correlational analyses relating cortisol, aldosterone, NT-proBNP and hs-CRP with metabolic, inflammatory and endothelial risk factors are shown in Tables 3 and 4. These markers illustrated positive and significant correlations (all at P < 0.001) with systolic and diastolic BP, glucose, insulin, all assessed cytokines (TNF-α, IL-6,IL-23) and endothelial markers (ICAM, VCAM and p-selectin). However, NT-proBNP and hs-CRP displayed subtle correlation with TG and TC from the lipid profile that was only significant at P < 0.05. NT-pro BNP and hs-CRP were positively correlated with TG (r = 0.414; r = 0.301; P < 0.05), and with TC (r = 0.431; r = 0.298; P < 0.05). Interestingly, cortisol was only significantly correlated with TC (r = 0.216, at P < 0.05).

**Table 3:** Correlations between cortisol, aldosterone, NT-proBNP and hs-CRP and metabolic risk factors among studied groups

	Cortisol	Aldosterone	NT-proBNP	hs-CRP
Systolic BP	r = 0.926 P < 0.001	r = 0.925 P < 0.001	r = 0.889 P < 0.001	r = 0.948 P < 0.001
Diastolic BP	r = 0.769 P < 0.001	r = 0.74 P < 0.001	r = 0.805 P < 0.001	r = 0.774 P < 0.001
Glucose (mg/dl)	r = 0.891 P < 0.001	r = 0.885 P < 0.001	r = 0.89 P < 0.001	r = 0.916 P < 0.001
Insulin (µIU/ml)	r = 0.963 P < 0.001	r = 0.974 P < 0.001	r = 0.856 P < 0.001	r = 0.957 P < 0.001
TG (mg/dl)	*NS*	*NS*	r = 0.414 P < 0.05	r = 0.301 P < 0.05
TC (mg/dl)	r = 0.216 P < 0.05	*NS*	r = 0.431 P < 0.05	r = 0.298 P < 0.05
LDL-C (mg/dl)	*NS*	*NS*	r = 0.438 P < 0.05	r = 0.302 <0.05
HDL-C (mg/dl)	*NS*	*NS*	*NS*	*NS*

Values are Pearson’s correlations using SPSS program

**Table 4:** Correlations between cortisol, aldosterone, NT-pro BNP and CRP on inflammatory and endothelial markers among studied groups

	Cortisol	Aldosterone	NT-pro BNP	hs-CRP
TNF-α	r = 0.964 P < 0.001	r = 0.971 P < 0.001	r = 0.89 P < 0.001	r = 0.992 P < 0.001
IL-6	r = 0.963 P < 0.001	r = 0.985 P < 0.001	r = 0.861 P < 0.001	r = 0.982 P < 0.001
IL-23	r = 0.83 P < 0.001	r = 0.902 P < 0.001	r = 0.707 P < 0.001	r = 0.899 P < 0.001
hs-CRP	*r* *=* *0.947* *P* *<* *0.001*	*0.975* *P* *<* *0.001*	*0.881* *P* *<* *0.001*	*–*
ICAM	r = 0.943 P < 0.001	r = 0.95 P < 0.001	r = 0.892 P < 0.001	r = 0.979 P < 0.001
VCAM	r = 0.986 P < 0.001	r = 0.985 P < 0.001	r = 0.856 P < 0.001	r = 0.961 P < 0.001
p-selectin	r = 0.986 P < 0.001	r = 0.973 P < 0.001	r = 0.874 P < 0.001	r = 0.967 P < 0.001

Values are Pearson’s correlations using SPSS program

**Fig. 2:**
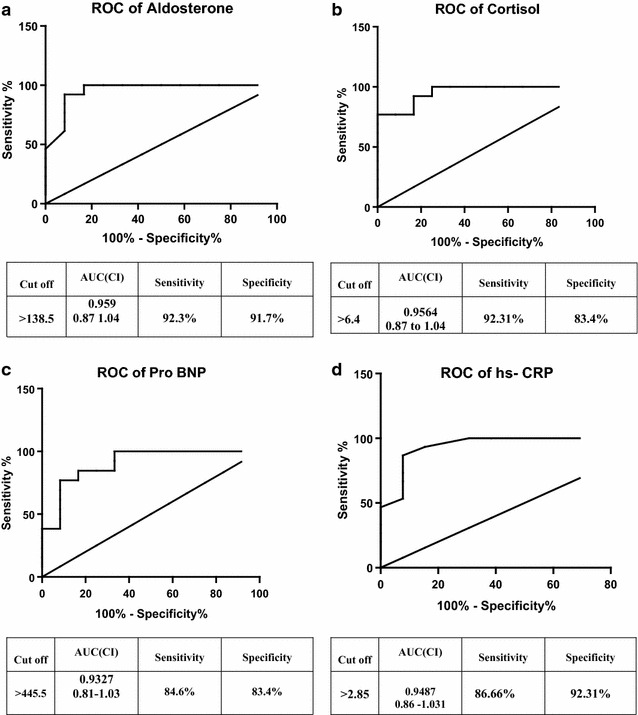
ROC *curves* panel of aldosterone, cortisol, NT-pro BNP and hs-CRP; **a** illustrates ROC of aldosterone; **b** illustrates ROC of cortisol; **c** illustrates ROC of NT-pro BNP, and **d** illustrates ROC of hs-CRP

The sensitivity and specificity of the assessed factors; cortisol, aldosterone, NT-pro BNP, and hs-CRP in predicting hypertensive-induced cardiovascular risk was illustrated via the Receiver Operating curves (Fig. 2a–d). The ROC analysis was applied between obese NT and pre-HT patients and showed that there was a high sensitivity and specificity for the predictive validity of aldosterone (92.307 % sensitivity and 91.7 % specificity; best cut off value >138.5 ng/dl; Fig. 2a), cortisol (92.307 % sensitivity and 83.4 %, specificity, best cut off value >6.4 ng/dl; Fig. 2b), NT-pro BNP (84.61 % sensitivity and 83.4 % specificity; best cut off value >445.5 pg/ml; Fig. 2c) in differentiating between NT group vs pre-HT metabolic syndrome groups. Interestingly, hs-CRP had the highest specificity (92.31 %), but sensitivity of 86.66 % and the best cut off value of >2.85 (mg/L), as shown in Fig. 2d.

## Discussion

In recent years, a considerable attention has been proposed on the involvement of HPA axis stimulation and secretion of adrenal hormones; aldosterone, cortisol in metabolic syndrome. Thus, data on the effects of adrenal hormones on myocardium, hypertension and its cardiovascular complications, as well as vascular endothelium, is being accumulated in adult population; however that in pediatrics remains scarce.

The current study presents a significant positive association of plasma aldosterone, and cortisol levels with obesity and elements of metabolic syndrome and their effect on immune-inflammatory markers according to the increased stage of hypertension in a pediatric cohort. This ensued in parallel with the escalating levels of adrenal hormones and NT-pro BNP levels in obese normotensive, pre hypertensive and stage 1 hypertensive patients. The correlation of the adrenal hormones and NT-pro BNP with the elements of MS and their utility as prognostic measures is the focus of the current study.

A number of studies demonstrated a greater responsiveness and hyperactivity of the HPA axis to various stimuli in patients with abdominal obesity exhibiting association with attenuated negative feedback in the HPA axis [9]. Certain mechanisms are implied attempting to describe the body fat mass impact on secretion of aldosterone and cortisol [10]. The latter refers to the direct effect of cortisol due to an increase in hepatic production of angiotensinogen, binding MC receptor, and a rise in vascular reactivity. In addition, the high flux of free fatty acids triggers a neuroendocrine reflex, resulting in the higher circulating levels of cortisol [3] notoriously linked to several cardiovascular dysfunctions including hypertension [4]. Since both the HPA axis and the sympathetic-adrenomedullary system are activated by psychological stress, aldosterone may be released due to psychological stress, that is reported to coincide with obesity states, providing an additional link between negative affective states and cardiovascular health. Early studies on stress characterized pathological consequences of excess mineralocorticoid activity referring to high blood pressure and evidence of myocardial fibrosis and necrosis [10].

AT-1 receptors mediate angiotensin-II induced stimulation of aldosterone release which is reported to be attributed to metabolic distortions associated to insulin resistance, including hyperglycemia, dyslipidemia and hypertension; events that are caused by acceleration of the atherosclerotic process and endothelial dysfunction [11]. This explains the current increase of aldosterone levels in the different obese groups, associated with the increased extent of hypertension and rationalizes the current positive correlation of aldosterone with glucose, insulin and HOMA-index, and currently assessed endothelial markers, ICAM, VCAM and p-selectin. Interestingly, no significant association was reported between aldosterone and all parameters of lipid panel.

With regard to the reported increase in HOMA-index, the surrogate marker of insulin resistance, studies in humans have shown that obesity is attributed to aldosterone increased production is correlated with increased insulin resistance, nonaligned with body mass index [12]. The findings above coincide with our results. Moreover, an elevated mineralocorticoid activity is reported to be one of the hypertension inducing mechanisms accounted for obesity [13].

Moreover, according to the pre-clinical studies, aldosterone induces the expression of the transcription factor, nuclear factor-kappa B (NF-κB) and genes responsible for inflammation, fibrosis, and atherosclerosis [14]. While experimental studies reported the reduction of atherosclerosis upon MR blockade [15], other clinical studies revealed that aldosterone infusion raised circulating IL-6 levels in contrast to spironolactone, which blocked IL-6 accompanied with administration of angiotensin II [16]. These evidences explain the current association of hyperaldosteronism and CRP, adipocytokine release of TNF-α, IL-6 & IL-23 as well as adhesion endothelial markers; ICAM and VCAM and p-selectin.

Based on the similarities between cortisol and aldosterone in response to HPA axis activation, it is assumed that some inducing factors engaged in dysregulation of both aldosterone and cortisol.

The current findings reported a significant decrease in cortisol in NT-obese group, compared to their control counterparts. However, with further elevation in BP in pre-HT and stage 1 HT groups cortisol was accordingly elevated.

Several studies have been conducted here and elsewhere investigating the link between cortisol and obesity; yet, the correlation between obesity and cortisol levels exhibits contradicting results and remains a subject of debate.

Indeed, low circulating cortisol concentrations have been measured in obese individuals where enhanced excretion of urinary free cortisol was found in those with MS [17], which assumingly may be referred to increased peripheral metabolism of cortisol. Contrariwise, a study that investigated the impact of cortisol on both body fat distribution and total fat in obese females did not support the previous finding [18]. As a matter of interest, cortisol clearance is likely to correlate negatively with insulin sensitivity, where such a correlation does not depend on body fat [19]. There are also numerous studies suggesting that glucocorticoids affect the differentiation and proliferation of human adipocytes with more receptors in visceral than in sc adipose tissue. In addition, glucocorticoids are reported to relocate adiposity from peripheral to central depots, increase the size and number of fat cells, and promote lipolysis with the release of free fatty acids into the circulation [20]. Another study also demonstrated the positive relation of excessive cortisol secretion to high intraabdominal fat distribution [21].

Being evident in about 80 % of adult patients and in almost 50 % in pediatrics and adolescent individuals, hypertension is one of the most significant factors of hypercortisolism. A lot of documented data suggested a link between cortisol and systolic and diastolic BP levels [18, 21]. This correlation is assumingly related to the impact of stress associated with the activation of the HPA axis and sympathetic nervous system. The above is predetermined by the fact that patients with MS and hypertension are reported to have more elevated urine levels of both cortisol and catecholamine metabolites in contrast to healthy individuals [22]. An increased responsiveness to vasoconstrictors, viz. ET-1, together with a reduced vasodilator production viz. NO is likely to be another mechanism by which glucocorticoids enhance BP. In addition, the endothelin system is activated in hypercortisolism, resulting in elevated plasma ET-1 levels that may take part in the pathogenesis of early atherosclerosis in such a disorder [23]. MS, hyperinsulinemia and insulin resistance are suggested to trigger ET-1 release which later on promotes renal injury and creates another pathway to hypertension [24]. No change or a slight rise in BP in normal subjects is observed as insulin exerts both; pressor (via enhanced sympathetic neural flow) and depressor (through vasodilatation) effects. On the other hand, in insulin resistance-hyperinsulinemia state, an imbalance between these two effects occurs [25].

These findings coincide partly with our results, as the current hypercortisolism in both obese pre-HT and HT groups is associated with concomitant derangements of glucose and insulin homeostasis, but failed to associate with any of the assessed lipid panel parameters. This is further confirmed by the significant positive correlation between cortisol and both glucose and insulin levels and the subtle, insignificant correlations with all parameters of the lipid profile. The latter finding contradicts with previous studies that found hypertriglyceridemia and low HDL-C were associated with increased cortisol, in both hypercortisolism and metabolic syndrome [26, 27]. The latter studies reported an association of hypercortisolism with elevated fasting blood glucose levels, especially with patients with metabolic syndrome. The correlation between fasting hyperglycemia and cortisol is caused by the glucocorticoid outcomes on hepatic gluconeogenesis and insulin secretion.

In the current study, an increased serum overnight cortisol level is likewise linked to insulin resistance, which in its turn is assessed against homeostasis model assessment. Thus, the following finding complies with in vivo and in vitro data proving that glucocorticoids regulate insulin secretion while higher cortisol concentrations are attributed to a reduced insulin secretion. Besides, a study conducted in obese children with- or without insulin resistance (homeostasis model assessment >4 or ≤4, respectively) demonstrated that body weight reduction contributed to lowering both cortisol levels and insulin resistance in the insulin-resistant group. However, children without insulin resistance did not manifest the same results [28].

In our study, obese subjects with higher BP displayed higher NT-pro BNP levels than non-obese individuals, which may have an impact on their susceptibility to hypertension and consequent cardiovascular disorders [29, 30]. Controversial findings on the levels of BNPs in several cardiac settings are reported in the literature. Unlike the findings of Mehra et al. [31] reporting decreased BNP in obese individuals with heart failure, our obese patients revealed higher NT-proBNP concentrations which are further increased with elevations in blood pressure. Our findings align with the results from another study which reported equivalent concentrations of increased A-type natriuretic peptide and BNP in obese normo- pre-hypertensive and hypertensive individuals [32].

Previous studies, on White, Asian and African American populations, have demonstrated an eloquent transposed relation between BNP levels and both prevalent metabolic syndrome and individual components of metabolic syndrome (obesity in specific) [33].

Moreover, BNP has been hypothesized to affect lipid and glucose metabolism through more direct pathways, as it may raise insulin levels, as well as glucagon secretion, instigating both lipolysis and release of triacylglycerols from adipose tissue [33].

A variety of factors other than myocardial stretch have been shown to stimulate secretion of BNP, such as myocardial ischemia, endocrine and paracrine factors such as endothelin, angiotensin II, and TNF-α. In the latest report on type 1 diabetes, TNF-α was postulated as a key molecule for the elevation of BNP and was demonstrated to be a key player in the development of diabetic complications [34]. These findings agree with the current positive correlation of NT-pro BNP and TNF-α. Moreover, the current data are confirmatory to the findings of Beygui et al. [35] for patients with high pro BNP levels showing higher cortisol and aldosterone levels.

These findings provide evidence that the addition of aldosterone and cortisol measurements could be used as an adjunct to NT-proBNP levels to define a subgroup of patients with very high risk of coronary heart disease. Moreover, the ROC analyses performed in the current study showed that there was a high sensitivity and specificity for the predictive validity of cortisol (100 % sensitivity and 92.31 % specificity), aldosterone (100 % sensitivity and 92.32 % specificity), NT-proBNP (100 % sensitivity and 92.308 % specificity) in differentiating between pre-HT group vs HT metabolic syndrome groups.

Concerning our secondary aim to assess the endothelial functions and their surrogate markers of immune-inflammatory endpoints in the tested groups, we found that metabolic syndrome in pediatrics is characterized by a low grade inflammatory state and is associated with an increase in immune-inflammatory mediators; hs-CRP and cytokines; TNF-α, IL-6 and IL-23, markers that are progressively elevated with increased stage of hypertension. These findings correspond with elevations of adrenal hormones; cortisol and aldosterone. However, only TNF-α and CRP showed a positive, significant correlation with TG,TC and LDL-C, while IL-6 and IL-23 failed to achieve significance.

In response to this elevated plasma level of the inflammatory cascade and LDL oxidation, a concomitant flux of adhesion factors; ICAM, VCAM and p-selectin are released by endothelial cells and are theorized to correlate with impaired vascular reactivity. Furthermore, the correlation analysis revealed significant positive correlation between endothelial markers; ICAM, VCAM and p-selectin; and each of systolic and diastolic BP, HOMA-index, all lipid profile except HDL-C and proinflammatory cytokines; TNF-α, IL-6, IL-23 and hsCRP.

Moreover, as explained in a study of Taddei et al. [36] on hypertensive patients’ offspring, such display of endothelial dysfunction is not only linked to cardiovascular disease but may also precede its development. Hence, although the study subjects were normotensive, they exhibited endothelial dysfunction. Interestingly, another study reported appearance of endothelial dysfunction in symptom-free children and young adults at high risk for atherosclerosis [37]. The contributing pathophysiologic mechanism include low NO bioavailability, upregulating VCAM-1 in the endothelial cell layer by inducing NF-κB expression [38], TNF-α, CRP, oxidized LDL and its receptor-1 (LOX-1). The expression of VCAM-1, ICAM-1, and p-selectin plays a role in triggering the inflammatory process and has been indicated as a possible link of a low-grade chronic inflammatory process to atherosclerosis and various endocrine disorders. VCAM-1 binds monocytes and T lymphocytes, which is the first stage of inflammatory cells invasion in the vessel wall [39].

In another study of Prazny et al. [40], increased ICAM-1 concentration was reported in patients with hypercortisolism, reflecting the induced endothelial dysfunction. It was shown that ICAM-1 was considerably higher in patients with increased cholesterol levels compared to those with increased BMI. The control group displayed higher ICAM-1 concentration in subjects with higher BMI. Besides, in the control subjects, ICAM-1 and p-selectin levels were strongly correlated with triglycerides. The findings above also demonstrate the link between endothelial dysfunction, obesity and lipid metabolism parameters under normal conditions. These findings are in alignment with ours, as VCAM is only endothelial marker that failed to correlate with our assessed lipid profile.

To this end and due to the key role aldosterone and cortisol play, both deserve consideration to be included as the primary screening target in metabolic syndrome patients, and earlier in patients with pre-HT and stage 1 HT patients for preventing CV complications. Moreover, the integration of these markers may increase the prognostic and diagnostic sensitivity and specificity and used for the risk stratification of metabolic syndrome patients and hopefully would aid in the prevention of development of cardiovascular diseases. However, the fact that this study is conducted in pediatrics limits the application of the results to pediatrics only. Further studies on adult cohorts are warranted to confirm its applicability on the adult population.

### Limitation of this study

The authors would like to acknowledge the limitations of the current study, which are expressed as follows: the first limitation concerns the small number of patients included in each subgroup in this pilot study, due to difficulty in recruitment and follow up with patients; this small sample size could be enlarged in further studies. The second limitation is the failure to include parallel lean cohorts with pre hypertension and overt hypertension for comparative purposes. Moreover, an extension of this study with a longer period of patient recruitment is warranted.
